# Why is clinical decision making not always efficient?

**DOI:** 10.1590/2177-6709.25.4.007-008.edt

**Published:** 2020

**Authors:** Flavia Artese

**Affiliations:** 1 Universidade do Estado do Rio de Janeiro, Departamento de Odontologia Preventiva e Comunitária (Rio de Janeiro/RJ, Brazil).

Herbert Simon, an American social scientist, described, in 1947, in his book *‘Administrative Behavior’*, the theory of decision-making. The main purpose of his work was to understand the cognitive and behavioral mechanisms that human beings use to make choices. Briefly, what Simon explains is that to make an efficient decision three steps are necessary: (*a*) to identify and list all alternatives; (*b*) to determine all consequences resulting from each alternative; and (*c*) to compare the precision and efficiency of each one of these consequences.

The process of converting all information about a problem into a decision is called judgement, which can be of an analytic or intuitive nature. The level of complexity of a problem influences the kind of judgement, which can be under certainty, when one single solution is possible; under risk, when few outcomes exist, and under uncertainty, when many outcomes are possible.^1^ We tend to use intuition to make judgements on complex problems, and as odd as it may seem, judgements in orthodontics are mostly under uncertainty.^2^

Two Israeli psychologists, Amos Tversky and Daniel Kahneman, researchers on behavioral psychology, described this intuitive judgement process in the 1970’s. They discovered that our brain, when facing complex decisions, that is, under uncertainty, tries to save energy. They called these mental shortcuts heuristics, a Greek word that means “I find”. Basically, it is defined as problem solving using a practical method that may not be optimal, perfect or rational. Nevertheless, it is sufficient to achieve an immediate result, quickly or by approximation.

Even though we proclaim evidence-based practice, which consists of the well-known tripod of the best evidence, professional experience and patient’s needs, there is still resistance in orthodontics. Apart from judgements under uncertainty, we still suffer from the lack of clear limits for what is considered a finished orthodontic treatment. It can vary from the simple alignment of teeth, with a cosmetic approach, to the ideal objectives, which include function, esthetics, tissue health and stability, with an approach focusing on health as well as beauty.

In this scenario, it is not hard to understand that some heuristic processes may happen in clinical decision-making. Heuristics of availability is the most frequent option to be remembered when solving a problem. Heuristics of representativity is a cognitive bias in which a choice is based on the chance of an event occurring due to the similarity to other past events already known. Both are extremely used in marketing. In this manner, our specialty has a strong tendency to choose appliances and techniques rather than an analytic process of decision-making. And this may probably explain why we have witnessed many trends of appliances and techniques, that at a certain point end up being circumscribed to their specific niches.^3^

Orthodontic practice combines science and operatory skills, which demand good professional education (including continued education) and manual dexterity, yet it has been assimilated by the world of artificial intelligence. This is not exclusive to orthodontics, since I believe most professions are feeling threatened by computer programs and robots. It may be possible that one part will be definitely performed by automation, exactly those in which decision-making is made under certainty and can thus be replaced by an operational algorithm. These facts, notwithstanding, as the level of complexity increases, our existence remains necessary. And I believe that in the future, orthodontic practice will be limited to high complexity only, demanding that professional education be even more profound to treat these cases efficiently. 

Therefore, it was with certain concern that I read the excellent paper by Theodore Eliades published in the AJO-DO this last July,^4^ in which he discusses the quality of the continuing education that we are receiving. In summary, the democratization of information with low evidence, wrapped in high technology, with commercial biases and with a market appeal, is the tendency of continued education of our specialty. Scientific evidence, a fundamental part of evidence-based practice, is shriveled in this scenario, and in a certain way, this conflicts with professional qualities that we will need even more in this future of high complexity only orthodontics. I am not against innovations, they are necessary and very welcome for our evolution, but they should not be confused with instant solutions or with strategies for patient enrollment. May they be part of the analytical decision-making process with the utmost concern of offering our patients not only a cosmetic treatment, but also all the complexity that a final orthodontic result demands: function, health, esthetics and stability. With no mental shortcuts.

Good reading!
